# Responses of the Graduate Program in Public Health to the covid-19

**DOI:** 10.11606/s1518-8787.2023057supl1ed

**Published:** 2023-05-11

**Authors:** Aylene Bousquat, José Leopoldo Ferreira Antunes

**Affiliations:** I Universidade de São Paulo Faculdade de Saúde Pública Departamento de Política, Gestão e Saúde São Paulo SP Brasil Universidade de São Paulo. Faculdade de Saúde Pública. Departamento de Política, Gestão e Saúde. São Paulo, SP, Brasil; II Universidade de São Paulo Faculdade de Saúde Pública Departamento de Epidemiologia São Paulo SP Brasil Universidade de São Paulo. Faculdade de Saúde Pública. Departamento de Epidemiologia. São Paulo, SP, Brasil

The covid-19 pandemic has required a rapid and incisive response from researchers in the most diverse fields of knowledge worldwide. Science has been challenged to change its rhythm and its time and to demonstrate the ability to subsidize policies to confront this unique health emergency.

Scientists’ voices have become prominent in public spaces worldwide. In the Brazilian scenario, scientists were even more important in opposing the denialist discourse and the necropolitics of the then federal government^1^. The Brazilian graduate system, despite its constant crises and underfunding, had an important contribution in this confrontation, quickly producing knowledge in the most diverse areas^7^.

The Graduate Program in Public Health (PPG-SP) of the School of Public Health of the University of São Paulo (USP) could not fail to contribute to this process. In its 53 years of existence, the PPG-SP has maintained its social commitment and sought to produce socially engaged and technical-scientifically qualified knowledge aimed to intervene in the national and international panorama of debates aimed at equating the national and global public health problems. With the emergence of the Covid-19 pandemic, the faculty and students quickly sought to contribute to the necessary confrontation, by producing knowledge and disseminating research-based information to the population.

This supplement presents a portion of the scientific production of professors, students, and graduates of the PPG-SP. In this issue, the reader will find a wide range of approaches, with different objects, points of view, and methodological approaches, which reflect the diversity and richness of the field of Collective Health in Brazil.

Shedding light on the persistent patterns of socio-spatial inequity in the municipality of São Paulo, Chiaravalloti-Neto et al. analyzed a cohort of almost 75,000 hospitalizations for covid-19 in São Paulo during the first year of the pandemic, demonstrating the importance of the place of residence in the risk of mortality from covid-19. These authors stress the need for broad public policies for the poorest areas, which allow to reduce these inequities.

Worldwide, health services and systems have been profoundly impacted. In addition to ensuring care for citizens with covid-19, the negative impact of covid-19 on the care of users with chronic conditions has been increasingly debated^8^. Unveiling these impacts is essential to develop health policies that reverse this situation as soon as possible. To that end, Cunha et al. analyzed the impact of the different phases of the covid-19 pandemic on hospitalizations for oral and oropharyngeal cancer in Brazil within the scope of the Unified Health System (SUS). Although the authors identified significant reductions in hospitalization after the pandemic emergence, these rates had not yet returned to pre-pandemic levels in 2021, indicating more bottlenecks for the system and worse prognoses for SUS users. Suda et al. analyzed the impact of the pandemic on the functioning of the Specialized Rehabilitation Centers (CER) in the SUS, both in the analysis of the variation in outpatient production and of the CER managers’ perceptions about the impacts of the pandemic on the units. The authors identified a severe impact of the covid-19 pandemic on CER: the impoundment of previous demands was compounded by the need to meet the numerous users with sequelae from covid-19, constituting a challenging picture.

Andrade et al. and Kamioka et al., in turn, estimated the SARS-CoV-2 virus antibodies seroprevalence in two populations with distinct characteristics: incarcerated women and schoolchildren aged four to 14 years living in the municipality of São Paulo. Notably, this last work expresses a very fruitful partnership with the Municipal Health Department of São Paulo.

The pandemic has also imposed changes in work processes, some of which have already been incorporated into everyday life. This supplement covers some of these changes. Freire et al. conducted a scope review on the use of telemedicine for patients with chronic diseases in long-term care actions (not covid-19) during pandemic, identifying that using telemedicine was positively related to the reduction of complications and the lack of need for physical displacement for care with expansion of assistance to rural areas. Important barriers were shown, some of them are: digital exclusion, sociocultural language barriers, and lack of accessibility of technological instruments for people with disabilities.

Also, when the use of telemedicine/telehealth is brought to the fore, an issue that emerges is telework and the need for its regulation. Castro et al. identified the legal norms published in 2020 and 2021 to directly or indirectly regulate telework in health in Brazil. They identified and analyzed more than 100 current legal norms on the regulation of telework in health. Despite the significant number, the regulation lacked provisions to guarantee both the defense of the worker’s and the patients’ rights or even the favorable conditions for carrying out the telework.

The impacts on sociability resulting from the necessary social isolation measures were experienced and faced in a very different way by the various portions of the Brazilian population. Cabral et al. examined the perceptions of adolescent students of a public school, living in a peripheral region of the municipality of São Paulo, with special focus on their experiences regarding education and sociability. The experience of remote learning was frustrating for the adolescents, without the daily and personalized monitoring of teachers. To the difficult or impossible access to the equipment and the absence of support from the schools is added the domestic environment that hindered the schooling process, especially for the girls, forced to assume more household chores. This article evinces how the measures to control the pandemic implemented in a fragmented manner and without support for poorer families negatively affected other spheres of life, specifically for poor young people.

Another health problem that has become even more prominent during the pandemic is the mental health of the population. In this supplement, the reader can approach this problem in the text by Rego et al. The authors hypothesized that people with mental health problems would be more likely to develop insomnia during the pandemic. In addition to confirming their hypothesis, the study indicated that insomnia can and should be understood as an important outcome for studies on the effects of unemployment and vulnerability on the population. Finally, Salles et al. discuss the incidence of symptoms suggestive of covid-19 among informal jewelry workers, who were already doing their work in their homes.

We are sure that this supplement will provide readers with important reflections on the covid-19 pandemic in Brazil. Finally, we cannot fail to thank the Coordination for the Improvement of Higher Education Personnel (Capes) which, through Proex, made possible the publication of this supplement.

Enjoy your reading.
