# Supplementary material for: Responses of the Graduate Program in Public Health to the covid-19
Source: Rev Saude Publica. 2023 May 11;57(Suppl 1):1s. doi: 10.11606/s1518-8787.2023057supl1ed (PMC10185310; doi:10.11606/s1518-8787.2023057supl1ed)
Supplement: Translation

# Translation: Respostas da Pós-Graduação em Saúde Pública à covid-19

**Authors:** Aylene Bousquat, José Leopoldo Ferreira Antunes

**Affiliations:** I Brasil Universidade de São Paulo. Faculdade de Saúde Pública. Departamento de Política, Gestão e Saúde. São Paulo, SP, Brasil; II Brasil Universidade de São Paulo. Faculdade de Saúde Pública. Departamento de Epidemiologia. São Paulo, SP, Brasil

A pandemia da covid-19 cobrou uma resposta rápida e incisiva dos pesquisadores das mais diversas áreas de conhecimento em todo o mundo. A ciência foi desafiada a mudar seu ritmo e seus tempos e a demonstrar capacidade de subsidiar as políticas de enfrentamento a esta emergência sanitária ímpar.

As vozes dos cientistas ganharam proeminência no espaço público em todo o mundo. No caso brasileiro, foram ainda mais importantes ao se contrapor ao discurso negacionista e à necropolítica do então governo federal^1^. O sistema de pós-graduação brasileiro, apesar de suas constantes crises e subfinanciamento, teve contribuição importante neste enfrentamento, produzindo rapidamente conhecimento nas mais diversas áreas^7^.

O Programa de Pós-Graduação em Saúde Pública (PPG-SP) da Faculdade de Saúde Pública da USP não poderia deixar de contribuir neste processo. Nos seus 53 anos de existência, o PPG-SP tem mantido seu compromisso social e buscado a produção de um saber socialmente engajado e técnico-cientificamente qualificado com o objetivo de intervir no panorama nacional e internacional dos debates que visam a equacionar os problemas da saúde pública nacional e mundial. Com o surgimento da pandemia da covid-19, rapidamente, o corpo docente e discente procurou contribuir para o necessário enfrentamento, tanto na produção de conhecimento, quanto na divulgação de informações cientificamente comprovadas para a população.

Este suplemento é dedicado a apresentar uma parcela da produção científica de docentes, discentes e egressos do PPG-SP. Neste número, o leitor encontrará uma ampla gama de abordagens, com distintos objetos, olhares e enfoques metodológicos, que refletem a diversidade e a riqueza do campo da saúde coletiva no Brasil.

Jogando luz nos persistentes padrões de inequidade socioespacial da capital paulista, Chiaravalloti-Neto et al. Analisaram uma coorte de quase 75.000 internações por covid-19 no município de São Paulo, durante o primeiro ano da pandemia, demonstrando a importância do local de moradia no risco de mortalidade por essa doença. Os autores reforçam a necessidade de amplas políticas públicas para as áreas mais pobres, que permitam reduzir essas inequidades.

Em todo o mundo, os serviços e sistemas de saúde foram profundamente impactados. Para além da garantia da atenção aos cidadãos com covid-19, cada vez mais tem sido debatido o impacto negativo da covid-19 no cuidado aos usuários com condições crônicas^8^. Desvelar esses impactos é central para a elaboração de políticas de saúde que revertam esse quadro com a maior brevidade possível. Nessa direção, Cunha et al. analisaram o impacto das diferentes fases da pandemia de covid-19 sobre as hospitalizações por câncer bucal e de orofaringe no Brasil, realizadas no âmbito do Sistema Único de Saúde (SUS). Apesar de os autores identificarem reduções importantes nas taxas de internações realizadas após o início da pandemia, estas ainda não haviam recuperado patamares anteriores à pandemia em 2021, indicando mais gargalos para o sistema e piores prognósticos para os usuários do SUS. Suda et al. analisaram o impacto da pandemia no funcionamento dos Centros Especializados em Reabilitação (CER) no SUS, tanto na análise da variação da produção ambulatorial como também das percepções de gestores de CER acerca dos impactos da pandemia nas unidades. Os autores identificaram severo impacto da pandemia de covid-19 nos CER: ao represamento de demandas prévias se somou a necessidade de atender os inúmeros usuários com sequelas de covid-19, configurando um quadro desafiador.

Andrade et al. e Kamioka et al., por sua vez, estimaram a seroprevalência de anticorpos do vírus SARS-CoV-2 em duas populações com características distintas: mulheres vivendo em restrição de liberdade e escolares de quatro a 14 anos de idade residentes no município de São Paulo, respectivamente. Vale ressaltar que este último trabalho expressa uma parceria bastante profícua com a Secretaria Municipal de Saúde de São Paulo.

A pandemia também impôs mudanças nos processos de trabalho, algumas das quais já se incorporaram no cotidiano. Neste suplemento, são abordadas algumas dessas mudanças. Freire et al. realizaram uma revisão de escopo sobre o uso da telemedicina para pacientes portadores de doenças crônicas em ações de cuidados continuados (não covid-19) no contexto da pandemia, identificando que a utilização da telemedicina esteve relacionada positivamente com a diminuição de complicações e a falta de necessidade de deslocamento físico para o atendimento com ampliação da assistência para áreas rurais. Barreiras importantes foram apresentadas, sendo as principais: a exclusão digital, barreiras socioculturais de idioma e falta de acessibilidade dos instrumentos tecnológicos para portadores de deficiência.

Adicionalmente, ao se trazer à tona o uso da telemedicina/telessaúde, uma questão que emerge é o teletrabalho e a necessidade de sua regulação. Castro et al. identificaram as normas jurídicas publicadas nos 2020 e 2021 para regular direta ou indiretamente o teletrabalho em saúde no Brasil. Mais de uma centena de normas jurídicas vigentes sobre a regulação do teletrabalho em saúde foram identificadas e analisadas. Apesar do expressivo número, foram identificadas ausências de dispositivos que regulassem o teletrabalho em saúde de modo a garantir tanto a defesa dos diretos do trabalhador, quanto dos pacientes, ou ainda as condições favoráveis à realização do teletrabalho em saúde.

Os impactos na sociabilidade decorrentes das necessárias medidas de isolamento social foram vivenciados e enfrentados de forma muita distintas pelas diversas parcelas da população brasileira. Cabral et al. examinaram as percepções de adolescentes estudantes de uma escola pública, moradores de uma região periférica da cidade de São Paulo, com especial enfoque sobre suas experiências quanto à educação e sociabilidade. A experiência do ensino remoto foi frustrante para os adolescentes, sem o acompanhamento cotidiano e personalizado de professores. Ao acesso difícil ou impossível aos dispositivos e à ausência de suporte das escolas é acrescido o ambiente doméstico que dificultou o processo de escolarização, em especial para as meninas, obrigadas a assumir mais tarefas de cuidado da casa e da família. Fica evidente neste artigo como as medidas de controle da pandemia implementadas de forma fragmentada e sem suporte às famílias mais pobres tiveram efeitos negativos em outras esferas da vida, em particular para jovens pobres.

Outro problema de saúde que ganhou ainda mais importância na pandemia é a saúde mental da população. Neste suplemento, o leitor poderá no texto de Rego et al. se aproximar desta problemática. Os autores partiram da hipótese de que as pessoas com problemas de saúde mental estariam mais propensas a desenvolver insônia durante a pandemia. Além da confirmação da hipótese, o estudo indicou que a insônia pode e deve ser entendida como um desfecho importante para estudos sobre efeitos do desemprego e vulnerabilidade na população. Por fim, Salles et al. discutem a incidência de sintomas sugestivos de covid-19 entre trabalhadores informais de joias, que já realizavam seu trabalho em suas casas.

Temos certeza que este suplemento proporcionará aos leitores reflexões importantes sobre a pandemia da covid-19 no nosso país. Por fim, não podemos deixar de agradecer à Coordenação de Aperfeiçoamento de Pessoal de Nível Superior (Capes) que, através do Proex, viabilizou a publicação deste suplemento.

Boa leitura.
